# A Trib2-p38 axis controls myeloid leukaemia cell cycle and stress response signalling

**DOI:** 10.1038/s41419-018-0467-3

**Published:** 2018-04-18

**Authors:** Mara Salomé, Aoife Magee, Krisha Yalla, Shahzya Chaudhury, Evgenia Sarrou, Ruaidhrí J Carmody, Karen Keeshan

**Affiliations:** 1 Paul O’Gorman Leukaemia Research Centre, Institute of Cancer Sciences, University of Glasgow, Glasgow, Scotland UK; 2 Centre for Immunobiology, Institute of Infection, Immunity and Inflammation, University of Glasgow, Glasgow, Scotland UK

## Abstract

Trib2 pseudokinase is involved in the etiology of a number of cancers including leukaemia, melanoma, ovarian, lung and liver cancer. Both high and low Trib2 expression levels correlate with different types of cancer. Elevated Trib2 expression has oncogenic properties in both leukaemia and lung cancer dependent on interactions with proteasome machinery proteins and degradation of transcription factors. Here, we demonstrated that *Trib2* deficiency conferred a growth and survival advantage both at steady state and in stress conditions in leukaemia cells. In response to stress, wild type leukaemia cells exited the cell cycle and underwent apoptosis. In contrast, *Trib2* deficient leukaemia cells continued to enter mitosis and survive. We showed that *Trib2* deficient leukaemia cells had defective MAPK p38 signalling, which associated with a reduced γ-H2Ax and Chk1 stress signalling response, and continued proliferation following stress, associated with inefficient activation of cell cycle inhibitors p21, p16 and p19. Furthermore, Trib2 deficient leukaemia cells were more resistant to chemotherapy than wild type leukaemia cells, having less apoptosis and continued propagation. *Trib2* re-expression or pharmacological activation of p38 in Trib2 deficient leukaemia cells sensitised the cells to chemotherapy-induced apoptosis comparable with wild type leukaemia cells. Our data provide evidence for a tumour suppressor role of Trib2 in myeloid leukaemia via activation of p38 stress signalling. This newly identified role indicates that Trib2 may counteract the propagation and chemotherapy resistance of leukaemia cells.

## Introduction

The Tribbles pseudokinases (Trib1, Trib2 and Trib3) are multifaceted signalling mediators controlling fundamental biological processes, including cell proliferation and survival, in both physiological and disease conditions^1^. *Tribbles* are inducible genes, modulated by a wide range of mitogens and stressors, and associated with downstream regulation of key signalling pathways, including AKT, ATF4, NF-kB and the MAPKs^2–6^. TRIB2 protein oscillates during cell cycle phases and induces the nuclear protein turnover of the dual specificity phosphatase and positive cell cycle mitotic regulator CDC25C^7^. Tribbles are newly recognised regulators of normal and malignant haemopoiesis^8,9^. Whilst *TRIB2* levels are low in myeloid cells at steady state^8^, TRIB2 has been shown to regulate activation and inflammatory functions of human monocytes and macrophages^3,10,11^. Recent investigations showed that Trib2 is required for normal T-cell and erythroid development^12,13^. Trib2 has been shown to interact with different MAPK kinases (MAPKK), such as MEK1 and MKK7, and to either promote or inhibit MAPK cascade activation in distinct cellular contexts^4,11^.

*Trib2* was first identified as an oncogene in acute myeloid leukaemia^14^. We have previously shown that Trib2 leukaemogenic potential relies on the ability to promote proteasomal dependent degradation of the tumour suppressor transcription factor CCAAT/enhancer-binding protein α (C/EBPα) and reported elevated *TRIB2* expression in a subset of human myeloid leukaemia patients with dysregulated C/EBPα profile and mixed myeloid/T-lymphoid phenotype^14,15^. Further studies have associated high and low levels of TRIB2 with leukaemia subtypes with distinct genetic backgrounds. *TRIB2* expression is positively associated with leukaemia patients that have *FLT3* mutated t(15;17) genetics^8^, and with patients with elevated BCL2 expression^46^, and is negatively associated with leukaemia patients that have *NPM1* and *FLT3* mutations^16^. It has also been shown that the absence of Trib2 accelerated NOTCH1-driven T-cell leukaemia development^12,17^. Both high and low *TRIB2* expression levels were shown to be associated with distinct human T-cell leukaemia phenotypes^12^.

The physiological role of Trib2 in myeloid leukaemia is not well understood. We previously showed that ectopic *Trib2* expression cooperates with Homeobox transcription factor Hoxa9 to accelerate myeloid leukaemia development in mice^18^. We and others have shown that expression of TRIB2 is driven by several transcription factors including NOTCH1^19,20^, TAL1^21^, PITX1^22^, MEIS1^23,24^ and E2F1^25^. The knockdown of TRIB2 in leukaemia cells led to leukaemia cell death^21,25^. However, low TRIB2 expression is associated with subgroups of myeloid leukaemia. It is not understood how the absence of Trib2 expression affects myeloid leukaemia.

Deregulation of the *HOX* genes occurs in ~70% of myeloid leukaemias. Indeed *HOXA9* alone is overexpressed in over 50% of acute myeloid leukaemia patients and correlates with poor prognostic outcome^26,27^. Here we used the oncofusion gene *NUP98/HOXA9* (NH9) as a deregulated HOX myeloid leukaemia model^28,29^ to investigate the effects of Trib2 deficiency in leukaemia cells. We showed that the absence of Trib2 does not impede the ability of NH9 to drive transformation. However, Trib2 deficiency enhanced myeloid leukaemia cell proliferation and survival in both steady state and stress conditions. Trib2 deficient leukaemia cells had impaired MAPK stress responses, evaded cell cycle checkpoint control mechanisms, and resisted chemotherapy-induced apoptosis. Our data identify Trib2 as a central regulator of p38-mediated stress signalling pathways and leukaemia cell cycle control.

## Results

### Trib2’s dispensability for NH9-initiated myeloid leukaemia

**Fig. 1:**
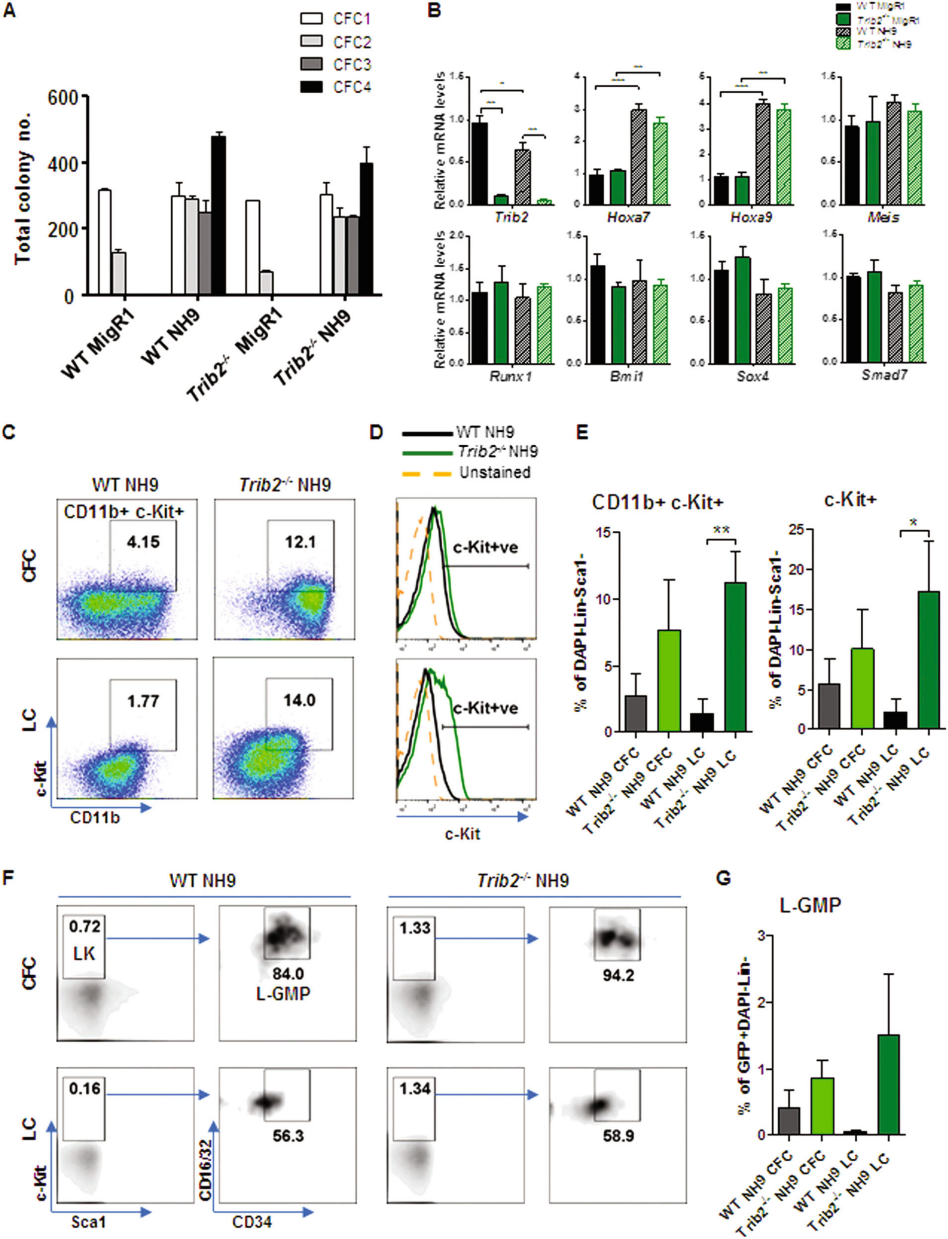
Trib2 is dispensable for NH9 initiated transformation of HSPCs. **a** CFC assay of WT and *Trib2*^*−/−*^ HSPCs retrovirally transduced with MigR1 or NH9 expressing vectors. Graph shows total colony number at CFC1-4. Data are mean of duplicate cultures ± SD; representative of 2 independent experiments. **b** Gene expression analysis in WT and *Trib2*^*−/−*^ HSPCs retrovirally transduced with MigR1 or NH9 at 48 h post transduction. Data are means of 3 biological samples (from 1 to 2 mice per biological replicate) ± SD and representative of 2 independent experiments. *P < 0.05, **P < 0.005, ***P < 0.001, using unpaired *t*-test. **c**, **d** Representative dot plots of the CD11b+c-Kit+ fraction (**c**) and c-Kit expression histograms (**d**) of WT and *Trib2*^*−/−*^ NH9 cells transformed by CFC assay (top panels) or LC condition (bottom panels), gated through GFP+DAPI-Lin(CD4, CD8, B220, Ter119)-Sca1- gates. **e** Graphed percentages are average of 3 independent measurements, as shown (**c**, **d**) ±SD. *P ≤ 0.05, **P < 0.005, using unpaired *t*-test. **f** Representative flow cytometry analyses identifying the L-GMP population in WT and *Trib2*^*−/−*^ NH9 cells transformed by CFC assay (top panels) or LC condition (bottom panels), gated through GFP+DAPI-Lin(CD3, CD4, CD8, CD19, B220, Ter119, Gr1)-CD127- and Sca1-c-Kit+(LK) gates. **g** Graph shows percentages of the L-GMP cells, as shown (**f**), in the GFP+DAPI-Lin-fraction. Data are mean of 3 independent measurements ± SD. *P ≤ 0.05, **P < 0.005, using unpaired *t*-test

The impact of Trib2 deficiency in myeloid leukaemia is not well understood. To address this, we investigated the ability of NH9 oncofusion to transform wild type (WT) and Trib2 knockout (*Trib2*^*−*^^*/−*^) haemopoietic stem and progenitor cells *(*HSPCs) (S1A). HSPCs were isolated from WT and *Trib2*^*−*^^*/−*^mice and transduced with NH9 or empty vector control MigR1 retrovirus (S1B). In vitro myeloid cell transformation was assessed by serial replating ability in a colony forming cell (CFC) assay (S1A). WT and *Trib2*^*−*^^*/−*^ MigR1 control groups failed to replate after the second round of CFC, whereas WT and *Trib2*^*−*^^*/−*^ NH9 HSPCs formed colonies up to the fourth replating indicative of cell transformation (Fig. 1a and S1C). Moreover, both WT and *Trib2*^*−*^^*/−*^ HSPCs transduced with NH9 and maintained in liquid culture (LC) conditions outgrew MigR1 controls and untransduced cells, as indicated by the increase in the fraction of GFP-expressing cells over time (S1D-E). To assess the self-renewal capability of NH9 in the absence of Trib2, a feature of leukaemic stem cells, we analysed the mRNA expression of *Hoxa9*, *Hoxa7*, *Meis1*, *Runx1*, *Bm1*, *Sox4* and *Smad7*^30–32^, in MigR1 or NH9 transduced WT and *Trib2*^*−*^^*/−*^ HSPCs 48 h post-transduction (Fig. 1b). Equal transduction levels and self-renewal profiles of NH9 expressing cells was confirmed by upregulation of the known target genes Hoxa7 and Hoxa9 in the WT and *Trib2*^*−*^^*/−*^ cells. Gene expression analysis of *Runx1*, *Bm1*, *Sox4* and *Smad7* revealed no differences between the WT and *Trib2*^*−*^^*/−*^ NH9 HSPCs or compared to MigR1 controls (Fig. 1b). Moreover, basal expression of *Hox* genes was equivalent between WT and *Trib2*^*−*^^*/−*^ MigR1 control cells. These data indicate that Trib2 is not required for NH9-mediated transformation of HSPCs into myeloid leukaemia cells. To confirm leukaemic stem cell properties of the WT and *Trib2*^*−*^^*/−*^ NH9 expressing cells we analysed leukaemic cell surface marker expression^33^by flow cytometry (Fig. 1c–g and S2A). Interestingly, the proportion of c-Kit+ cells, and the Lineage(Lin)-Sca1-CD11b+c-Kit+ fraction were higher in the Trib2 deficient cells compared to the WT NH9 cells (Fig. 1c–e). Similarly, Lin-Sca1-CD127-c-Kit+CD34+/lowCD16/32+ cells, previously identified as L-GMP (leukaemic granulocyte macrophage progenitors^34^), were increased in the *Trib2*^*−/−*^ NH9 compared to the WT NH9 cells (Fig. 1f, g and S2B). Overall these results suggest that Trib2 deficient cells retain higher leukaemic stem cell potential.

### Trib2 deficiency promotes the propagation and survival of leukaemia cells

**Fig. 2:**
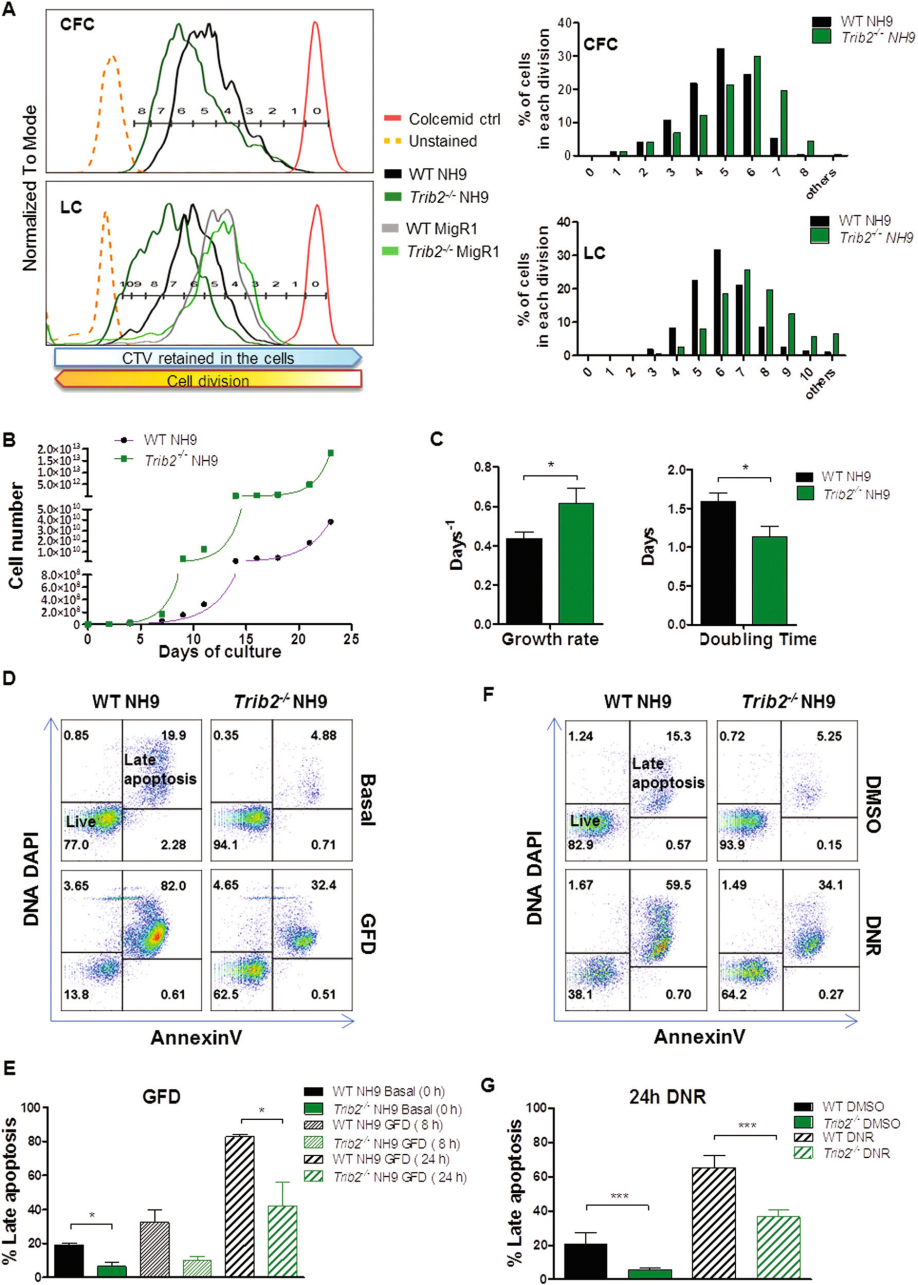
Trib2 deficiency accelerates cell proliferation promotes survival in NH9 leukaemic cells. **a** CTV assay of WT and *Trib2*^*-/-*^ NH9 cells derived from CFC3 (CFC, top) and CTV assay of WT and *Trib2*^*-/-*^ MigR1/NH9 cells derived from 2 weeks in LC, in presence of recombinant SCF, IL3, IL6 (LC, bottom). Samples were analysed by flow cytometry at 96 h after staining with the CTV dye, and gated through GFP+ gates. Undivided, stained control cells are shown in red (colcemid ctrl). Cell division gates are indicated and % of cells in each cell division for the transformed WT and *Trib2*^*-/-*^ NH9 cells is graphed. Data are representative of at least 3 independent experiments. **b** Growth curves (right) of WT and *Trib2*^*-/-*^ NH9 cell lines, grown in presence of IL3 (NH9 medium). In green and pink are the projected non-linear regression curves that best fit the exponential growth models of WT and *Trib2*^*-/-*^ NH9 cells, respectively (calculated with the GraphPad software). **c** Growth rate and doubling time of WT and *Trib2*^*-/-*^ NH9 cells, as measured in B. Data shown are means of 3 independent experiments ± SD. *P < 0.05, using unpaired *t*-test. **d**–**g** Flow cytometric analysis of apoptotic levels by means of AnnexinV/DNA DAPI staining in WT and *Trib2*^*-/-*^ NH9 cell lines after GFD (**d**) and 24 h DNR treatment (**f**). **f**, **g** Graphed percentages of Late Apoptotic cells (AnnexinV+/DNA DAPI+), at the indicated time points, as measured in (**d**) and (**f**), respectively. Data are representative of at least 3 independent experiments, graphs show mean ± SD. *P ≤ 0.05, **P < 0.005, ***P < 0.001, using unpaired *t*-test

To determine the impact of Trib2 deficiency on the growth properties of myeloid transformed cells, we compared the cell division using the viable dye cell trace violet (CTV) of MigR1 or NH9 transduced WT and *Trib2*^*−/−*^ HSPCs (from two independent transduction experiments) that had expanded by CFC assay (GFP sorted, Fig. 2a, top panels) or in liquid culture (LC) for 2 weeks (unsorted for GFP, Fig. 2a, bottom panels). Our results showed a higher number of cell divisions in the *Trib2*^*−/−*^ NH9 cells compared to the WT NH9 cells (Fig. 2a), indicating that Trib2 negatively regulates myeloid leukaemia cell proliferation. WT and *Trib2*^-/-^ NH9 transduced cells were expanded in LC for several months. Growth curves confirmed faster proliferation rate and significantly shorter doubling time in *Trib2*^*-/-*^ NH9 cells compared to WT NH9 cells (Fig. 2b, c). To assess the impact of Trib2 deficiency on myeloid leukaemia cell survival, we measured the level of apoptosis following growth factor deprivation (GFD) and myeloid leukaemia-based chemotherapy (daunorubicin (DNR)) in the WT and *Trib2*^*-/-*^ NH9 cells by flow cytometry using AnnexinV/DAPI staining (Fig. 2d–g). Our analyses showed that basal levels of apoptosis in *Trib2*^*-/-*^ NH9 cells were significantly lower compared to the WT NH9 cells (~5 vs. ~20% respectively, Fig. 2d–g 0 h GFD and DMSO treated samples). Following GFD or DNR treatment, *Trib2*^*-/-*^ NH9 cells exhibited lower levels of cell death compared to the WT NH9 cells (Fig. 2d, e 24 h GFD and Fig. 2f, g DNR treated samples). These data show that Trib2 deficiency promotes myeloid leukaemia cell proliferation and survival.

### Impaired MAPK activation in the absence of Trib2

**Fig. 3:**
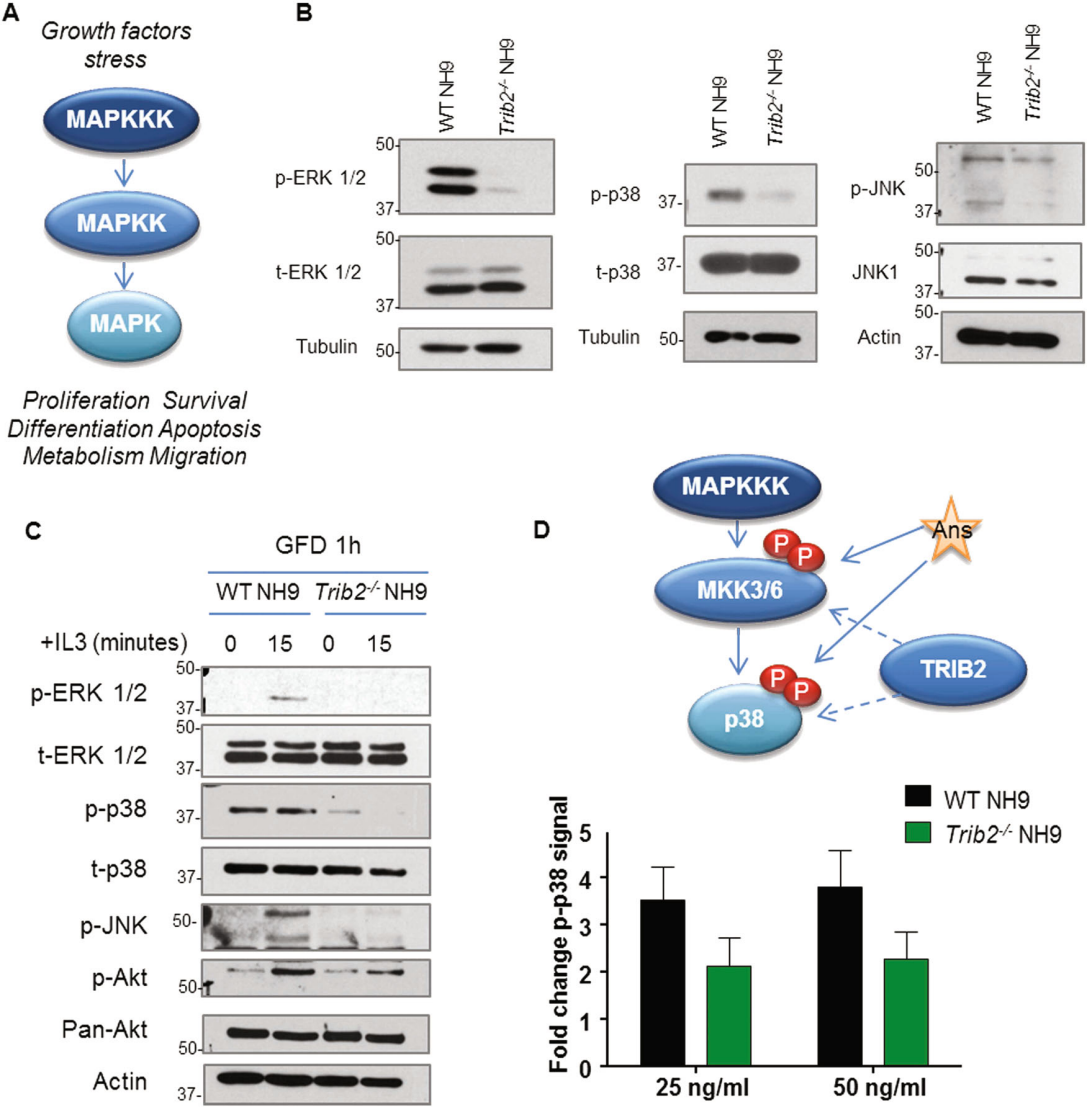
Trib2 deficient myeloid leukaemia cells have impaired MAPKs activation. **a** Schematic of MAPK signalling cascade. **b** Western blot analysis of MAPKs total and phosphorylated levels (ERK1-2 (Thr202/Tyr204), p38 (Thr180/Tyr182) and JNK (Thr183/Tyr185)) in WT and *Trib2*^*-/-*^ NH9 cells. Data are representative of at least 3 independent experiments. **c** Western blot analysis of MAPKs and AKT (Ser473) activation in WT and *Trib2*^*-/-*^ NH9 cells after 1 h of GFD, followed by re-stimulation with IL3-containing medium (NH9 medium) for 0 and 15 min. **d** Ans binds and activates the p38 signalling pathway. Model of TRIB2-mediated regulation of MKK3/6 or p38 kinases (top). The p-p38 signal was measured by flow cytometry following stimulation of the WT and *Trib2*^*-/-*^ NH9 cells with 25 and 50 ng/ml Ans, and normalised by DMSO treated control cells (bottom). Data shown is average of 2 independent experiments ± SD, the genotype significantly affects the result, *P* < 0.05 using two-way ANOVA

Trib2 modulates MAPK signalling^3,4,11,12^, and MAPKs are linked to cell proliferation and cancer^35,36^. Thus, we interrogated the activation status of the MAPKs ERK, p38 and JNK in WT and *Trib2*^*-/-*^ myeloid leukaemia cells. Western blot analyses showed that *Trib2*^*-/-*^ NH9 cells had lower levels of the phosphorylated (active) forms of ERK1-2 (Thr202/Tyr204, p-ERK), p38 (Thr180/Tyr182, p-p38) and JNK (Thr183/Tyr185, p-JNK), compared to the WT NH9 cells (Fig. 3b). WT and *Trib2*^*-/-*^ NH9 cells showed IL3-dependent growth (Fig. 2b), therefore we tested MAPK following GFD and re-stimulation with IL3-containing medium (NH9 medium). We showed that MAPKs phosphorylation was impaired in *Trib2*^*-/-*^ NH9 compared to WT NH9 thus indicating inefficient MAPK responses in *Trib2*^*-/-*^ NH9 (Fig. 3c). Conversely, the phosphorylation of AKT (Ser473) was similarly activated following IL3 stimulation in both WT and *Trib2*^*-/-*^ NH9 cells (Fig. 3c). We tested the activation of p38 in response to anisomycin (Ans), a pharmacological activator of the p38 and JNK pathway, that directly activates MKK6 and p38 kinases^37^ (Fig. 3d). The fold increase in p-p38 after Ans treatment measured by phosphoflow cytometry was reduced in the *Trib2*^*-/-*^ NH9 cells compared to the WT NH9 cell (Fig. 3d). These data showed that Trib2 is necessary for efficient activation of the p38 kinase. Overall, these results showed that Trib2 deficient myeloid leukaemia cells have compromised basal- and induced-MAPK signalling activation.

### Trib2 deficiency enables cell cycle progression following stress

**Fig. 4:**
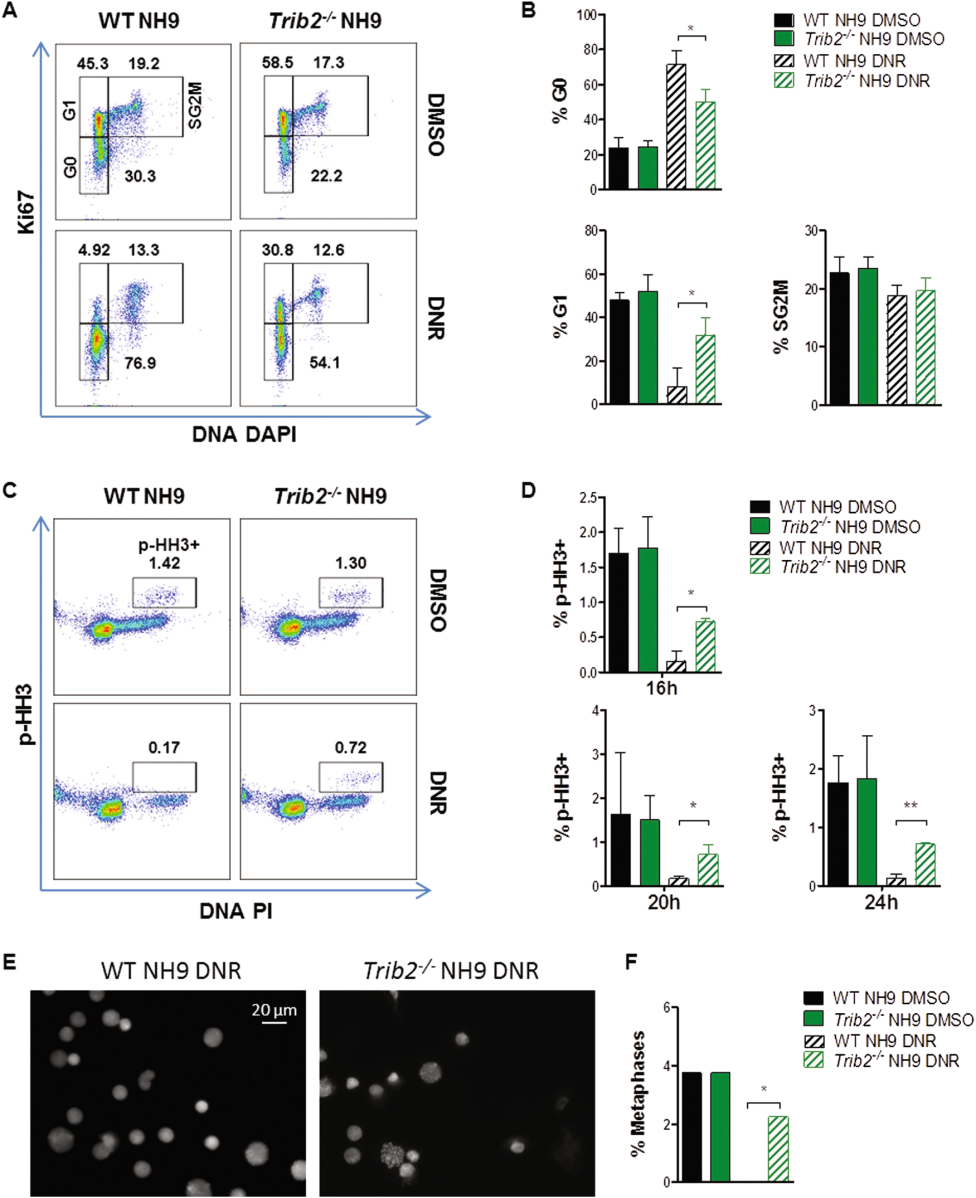
Trib2 deficient myeloid leukaemia cells have an impaired cell cycle stress response. **a** Cell cycle flow cytometric analysis by means of Ki67/DNA staining in WT and *Trib2*^*-/-*^ NH9 cells in response to DNR treatment. Plots are representative of 6 independent experiments with similar trend. **b** Graphed percentages of G0, G1 and SG2M fractions, as measured in (A). Data are means ± SD of 3 independent experiments. *P < 0.05, using unpaired *t*-test. **c** Flow cytometric analyses of the mitotic index in WT and *Trib2*^*-/-*^ NH9 cells after 24 h DNR treatment, as measured by p-HH3/PI DNA levels. **d** Graphed percentages, at the indicated time points, as measured (**c**). **e** Representative pictures of DAPI stained metaphase spreads from WT and *Trib2*^*-/-*^ NH9 cells after 24 h DNR treatment. **f** Graph showing percentages of mitotic spreads from WT and *Trib2*^*-/-*^ NH9 cells after 24 h DNR/DMSO treatment as measured **e**. Data are representative of at least 2 independent experiments, graphs show mean ± SD. *P < 0.05, **P < 0.005, using unpaired *t*-test

To gain further insight into the mechanism involved in the enhanced growth and survival of Trib2 deficient leukaemic cells, we assessed the cell cycle control and check point activation in response to genotoxic stress in WT and *Trib2*^*-/-*^ NH9 cells. Using Ki67/DNA DAPI flow cytometry staining, we assessed the cell cycle profile of WT and *Trib2*^*-/-*^ NH9 cells following DNR treatment. No significant difference in the cell cycle profiles was observed in untreated WT and *Trib2*^*-/-*^ NH9 cells, where the majority of the cells were in the G1 phase of the cell cycle (Fig. 4a, b). Following DNR treatment, the majority of WT NH9 cells were lost from the active G1, S, G2, M phases and with remaining cells in the GO quiescent phase of the cell cycle. In contrast, a significant proportion of *Trib2*^*-/-*^ NH9 cells were still cycling and with less cells in the GO quiescent state (Fig. 4a, b). We next assessed the proportion of cells in the mitotic phase (mitotic index) by p-HH3/DNA PI staining. In untreated cells, no significant differences were observed between the WT and *Trib2*^*-/-*^ NH9 cells. However, a significantly higher fraction of p-HH3+ cells was observed in *Trib2*^*-/-*^ NH9 cells compared to WT NH9 cells, following 16, 20 and 24 h post DNR treatment (Fig. 4c, d). Similarly, a higher fraction of p-HH3+ cells was detected upon GFD condition in *Trib2*^*-/-*^ NH9 (Figure S3). We analysed metaphase spreads of DNR-treated WT and *Trib2*^*-/-*^ NH9 cells by microscopy to validate the role of Trib2 in mitotic cell cycle control following stress stimuli. Similar to p-HH3 mitotic indices, there was an equivalent percentage of mitotic spreads in the untreated WT and *Trib2*^*-/-*^ NH9 cells (Fig. 4e, f). DNR treatment virtually abolished mitotic entry in the WT NH9 cells, as no mitotic spreads were observed in any field/slide analysed. Conversely, a significantly higher proportion of DNR-treated *Trib2*^*-/-*^ NH9 were in metaphase compared to WT NH9 cells (Fig. 4e, f). These data show that *Trib2*^*-/-*^ NH9 cells can actively cycle and undergo mitosis in the presence of stress. These results indicate a role for Trib2 in the prevention of cell cycle progression in stress conditions.

### Trib2 deficiency compromises stress response signalling in myeloid leukaemia cells

**Fig. 5:**
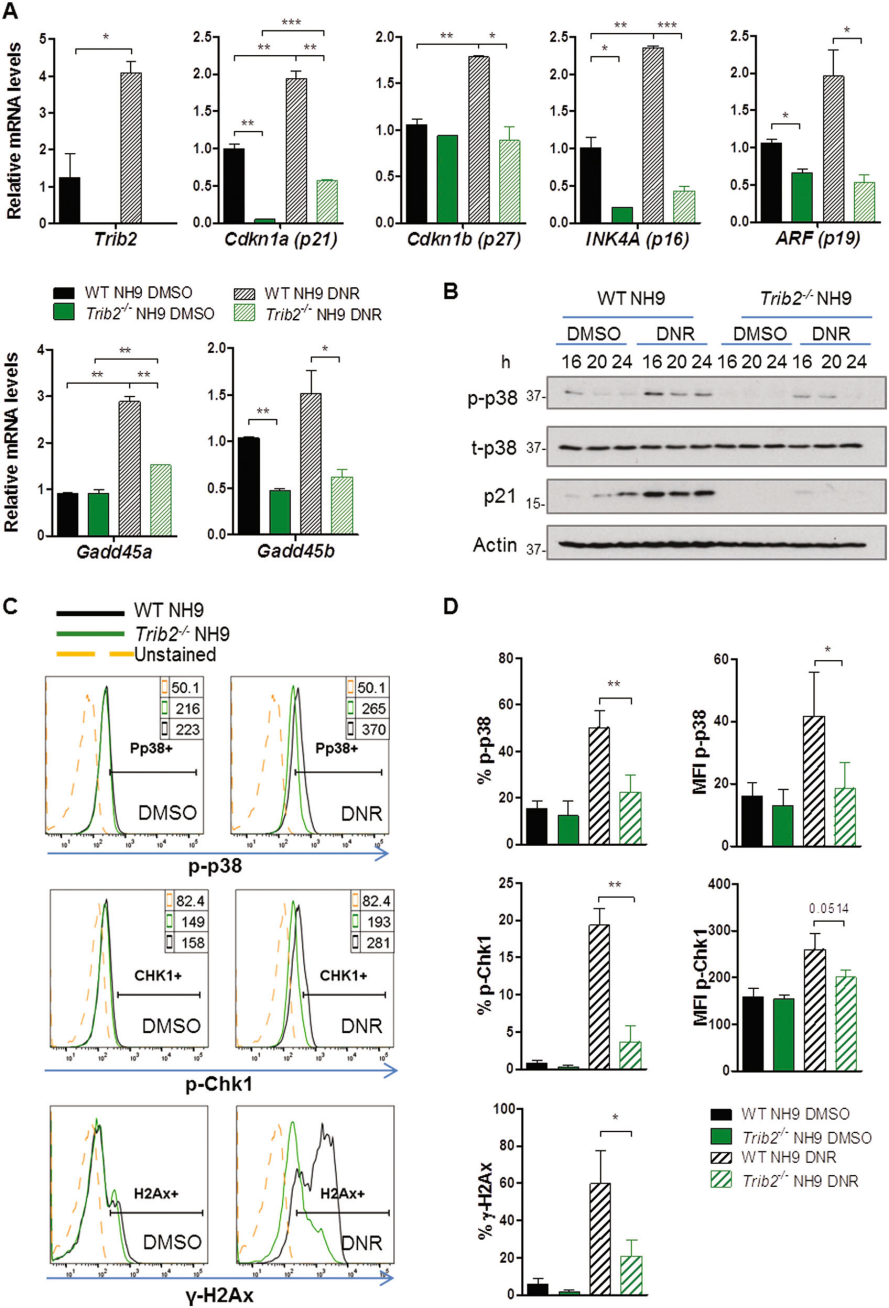
*Trib2* is required for activation of cell cycle inhibitors and check point regulators following drug treatment. **a** *Trib2*, *Cdkn1a* (p21), *Cdkn1*b (p27), *INK4A* (p16), *ARF* (p19), *Gadd45a* and *Gadd45b* relative mRNA levels in WT and *Trib2*^*-/-*^ NH9 cells after 16 h DNR treatment. Data are representative of 2 (p16 and *Gadd45a*) or 3 (*Trib2*, p21, p27, p19 and *Gadd45b*) independent experiments with similar trend, graphs show mean of technical replicates ± SD. *P < 0.05, **P < 0.005, ***P < 0.001 using unpaired *t*-test. **b** Western blot analysis of the indicated proteins in WT and *Trib2*^*-/-*^ NH9 cells in response to DNR treatment for the indicated time points. Data is representative of at least 3 independent experiments, with similar trend. **c** Flow cytometric analysis of p-p38, p-Chk1 and γH2Ax levels in the live fraction of WT and *Trib2*^*-/-*^ NH9 cells, after 24 h DNR treatment. Histograms are representative of at least 3 independent experiments, with similar trend. **d** Graphed percentages (**c**) show average ± SD of three independent measurements. MFI Median Fluorescent Intensity (indicated in the top right panel of the representative histograms). *P < 0.05, **P < 0.005, ***P < 0.001 using unpaired *t*-test

As we have demonstrated a role for Trib2 in leukaemia cell cycle control in response to stress, we hypothesized that Trib2 is an inducible component of the cellular stress response pathways, contributing to check point activation and survival or death decision. To address this, we assessed *Trib2* mRNA expression levels following GFD and DNR treatment. In WT NH9 cells, a significant upregulation of *Trib2* mRNA expression was observed in response to DNR treatment (Fig. 5a) and GFD (Figure S4A). Gene expression analyses of DNR treated WT and *Trib2*^*-/-*^ NH9 cells showed that the activation of cell cycle inhibitors and regulators, including *Cdkn1a* (p21), *Cdkn1b* (p27), *INK4A* (p16), *ARF (p19)* and *Gadd45a/b*, was abolished or significantly lowered in the absence of *Trib2*. Indeed, levels of *Cdkn1a* (p21), *INK4A* (p16), *ARF* (p19) and *Gadd45b* were lower in untreated *Trib2*^*-/-*^ NH9 cells compared to the WT NH9 cells (Fig. 5a). Similar results were observed following GFD (Figure S4A). Levels of *Cdk4*, a positive regulator of cell cycle progression, were higher in *Trib2*^*-/-*^ NH9 cells compared to the WT NH9 cells, at both steady state and upon GFD (Figure S4A), consistent with the highly proliferating phenotype of *Trib2*^*-/-*^ NH9 cells. In addition, *Trib2*^*-/-*^ NH9 cells failed to upregulate *Egr1* and *Nfatc4* (apoptotic mediators), *Jun* and *Fos* (stress response TFs downstream of MAPK signalling), mRNA in both vehicle control and DNR treated samples (Figure S4B). These results demonstrate that Trib2 expression is required in myeloid leukaemia cells for efficient activation of cell cycle check point genes and DNA damage response signalling pathways. Assessment of key cell cycle checkpoint regulator proteins by western blotting showed that induction of p-p38 and p21 was impaired after 16, 20 and 24 h DNR treatment in *Trib2*^*-/-*^ NH9 compared to WT NH9 cells. Importantly, the total protein levels of p38 level were unaffected (Fig. 5b). Furthermore, mRNA levels of several MAPKs genes, including *MAPK3* (ERK1), *MAPK9* (JNK2), *MAPK11* (p38β), *MAPK12* (p38γ), *MAPK13* (p38δ), and the *MKNK1* (MNK1), were significantly lower in *Trib2*^*-/-*^ NH9 cells in response to DNR treatment compared to the treated WT NH9 cells (Figure S4C). Reduced activation of the MAPK p-p38 was also confirmed by flow cytometry in WT and *Trib2*^*-/-*^ NH9 cells in response to DNR treatment (Fig. 5c, d). The fraction of p-p38+ cells and the MFI of p-p38 (which correlates with the number of p38 molecules activated per cell) were significantly lower in the *Trib2*^*-/-*^ NH9 treated cells compared to the WT NH9 cells. Levels of p-Chk1 and γ-H2Ax (Ser139), key regulators of DNA damage response signalling, were significantly reduced in *Trib2*^*-/-*^ NH9 treated cells compared to the WT NH9 treated cells (Fig. 5c, d). Together these data demonstrate a role for Trib2 as a major orchestrator of cell cycle check point and stress signalling pathways in leukaemia, required for efficient activation of p38, Chk1 and γ-H2Ax, and stress-induced p21 expression.

### Trib2 deficiency enables propagation of drug resistant leukaemic cells

**Fig. 6:**
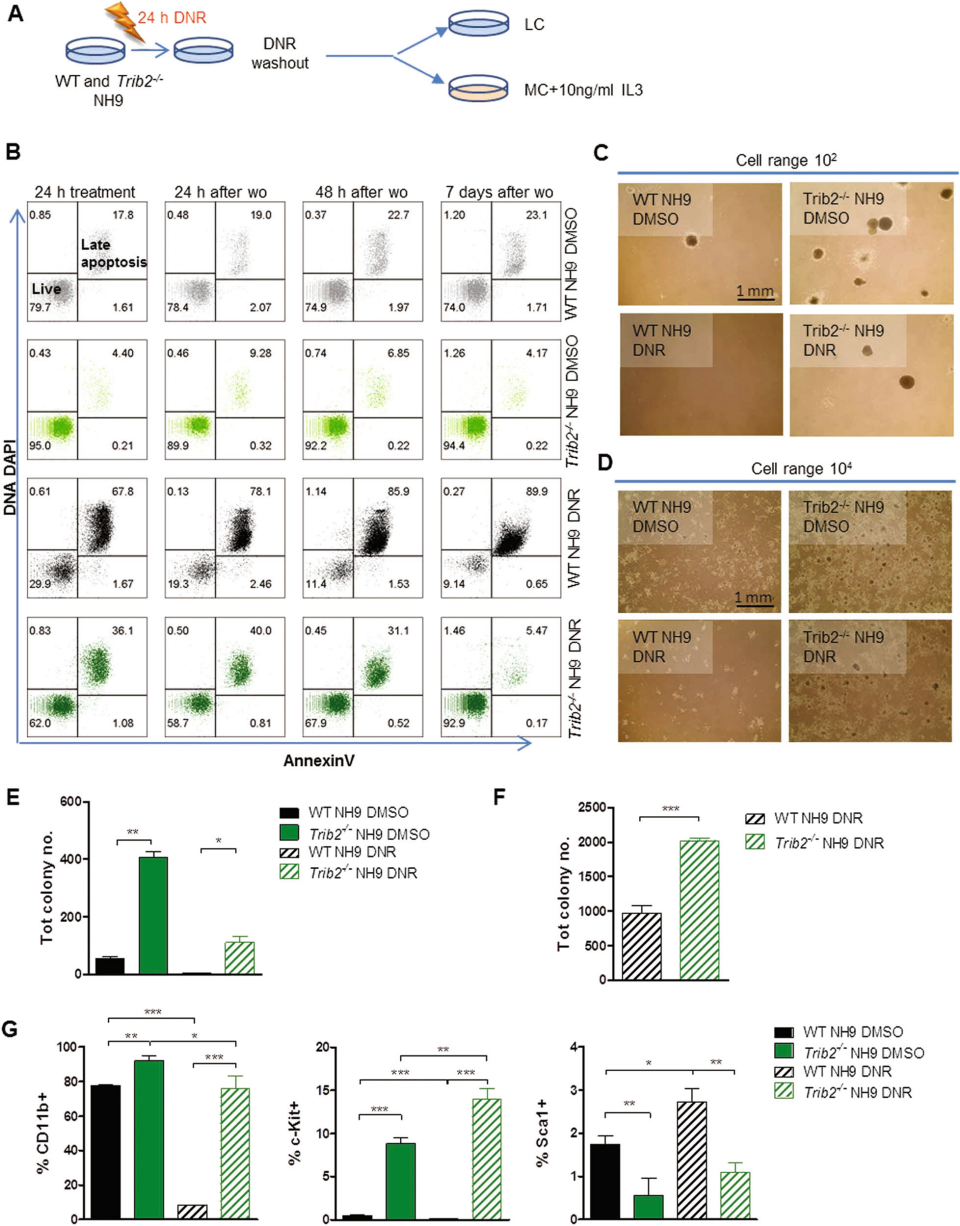
*Trib2*^*-/-*^ NH9 cells are more resistant to DNR-dependent apoptosis after removal of the drug. **a** Schematic showing experimental strategy of the DNR wo experiments in LC or IL3-supplemented MC. **b** Apoptosis analysis of the WT and *Trib2*^*-/-*^ NH9 cells after 24 h DMSO/DNR treatment, and at 24, 48 h and 7 days after drug wo. **c** Day 7 and **d** day 5 colony pictures (4×) of WT and *Trib2*^*-/-*^ NH9 samples treated with DMSO/DNR for 24 h and plated in MC, after wo, at the indicated cell range, in independent experiments. **e**, **f** Colony counts at day 7, as per experiment in (**c** and **d**), respectively. Graphs show mean of duplicate (**c**, **e**) or triplicates (**d**, **f**) cultures ± SD. *P < 0.05, **P < 0.005, ***P < 0.001 using unpaired *t*-test. **g** Flow cytometric analysis of the indicated myeloid and primitive surface markers of WT and *Trib2*^*-/-*^ NH9 cells in (**d**, **f**), grown in MC for 7 days, after removal of the drug. Graphs show mean of triplicate cultures ± SD. *P < 0.05, **P < 0.005, ***P < 0.001 using unpaired *t*-test

To test whether the inappropriate stress response of Trib2 deficient leukaemia cells results in the propagation of drug-resistant myeloid leukaemia cells, we performed DNR washout (wo) experiments. WT and *Trib2*^*-/-*^ NH9 cells were treated with the drug for 24 h, then washed and replated in either LC or CFC assays (Fig. 6a). *Trib2*^*-/-*^ NH9 cells displayed reduced apoptotic rate in LC, and higher clonogenic potential compared to the WT NH9 cells in CFC (Fig. 6b-f). Moreover, cell surface marker analyses revealed that drug resistant *Trib2*^*-/-*^ NH9 cells continued to express CD11b myeloid marker, higher levels of the immature marker c-Kit (Fig. 6g, left and central panels), and lower levels of the inflammatory surface marker Sca1 compared to WT NH9 cells in both untreated and drug resistant cells (Fig. 6g, right panel). These data indicate the Trib2 deficiency contributes to a drug resistant phenotype in myeloid leukaemia.

### Re-expression of *Trib2* reverts the drug resistant phenotype of Trib2-deficient myeloid leukaemia cells

**Fig. 7:**
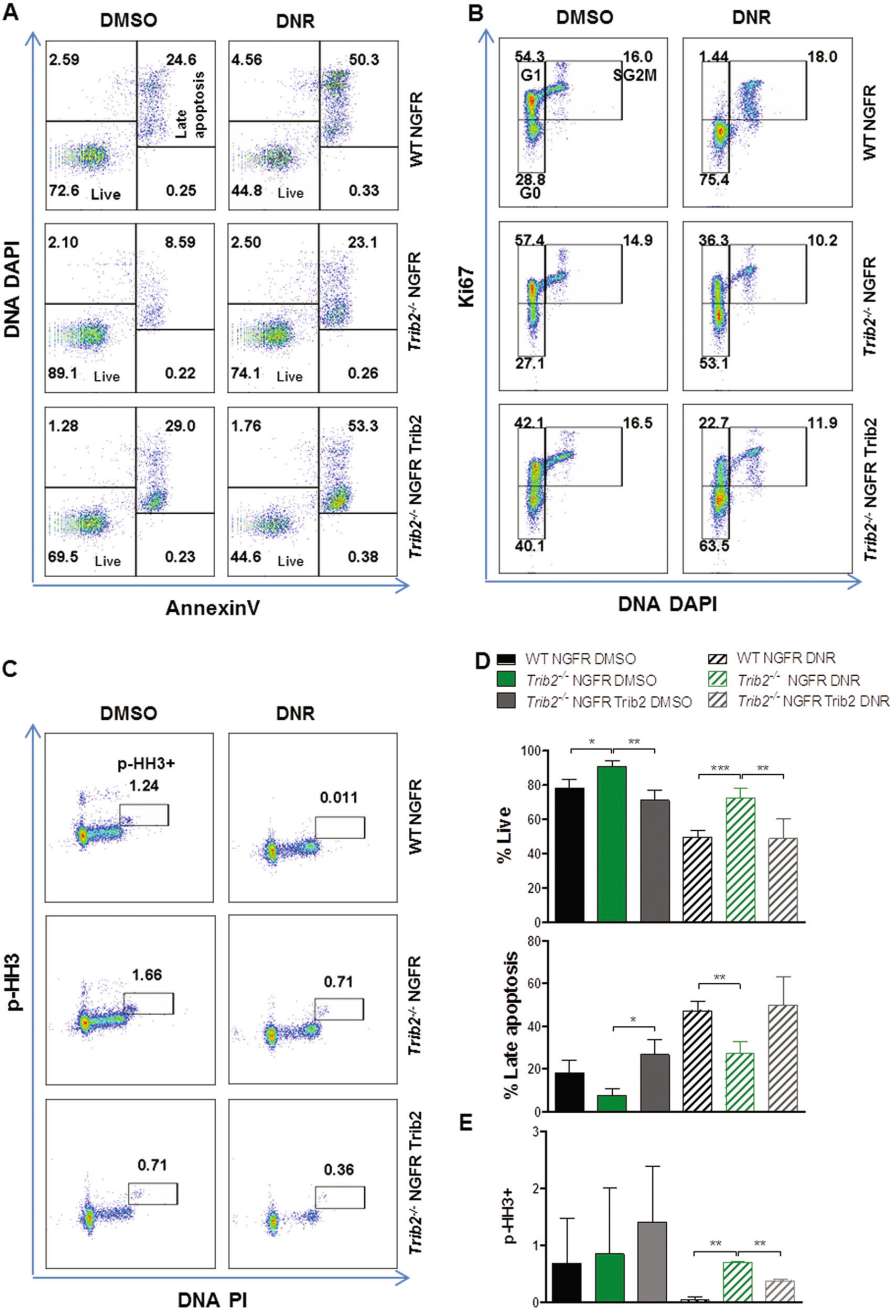
Re-introduction of *Trib2* in *Trib2*^*-/-*^ NH9 cells re-establishes an apoptotic and cell cycle response to drug treatment. Flow cytometric analysis of apoptotic levels by means of AnnexinV/DNA DAPI (**a**), Cell cycle Ki67/DNA DAPI (**b**), and mitotic index by means of p-HH3/DNA DAPI staining (**c**) in WT and *Trib2*^*-/-*^ NH9 MigR1 NGFR/NGFR Trib2 transduced cells, after 24 h DNR treatment. **d** Shows graphed percentages of Live (AnnexinV-/DNA DAPI-) and Late Apoptotic (AnnexinV+/DNA DAPI+) cells, as measured (**a**). Data are representative of 4 independent experiments, graphs show mean ± SD. *P < 0.05, **P < 0.005, ***P < 0.001, using unpaired *t*-test. **e** Graphed percentages of p-HH3+ cells, as measured (**c**). Data are representative of 2 independent experiments, graphs show mean ± SD. **P < 0.005 using unpaired *t*-test

To validate the drug resistant phenotype as a result of the Trib2 deficiency, we reintroduced Trib2 protein expression in the *Trib2*^*-/-*^ NH9 cells. WT and *Trib2*^*-/-*^ NH9 were retrovirally transduced with NGFR and NGFR Trib2, and*Trib2* mRNA or protein expression confirmed in NGFR+ sorted cells (Figure S5). NH9-expressing WT NGFR, *Trib2*^*-/-*^ NGFR, and *Trib2*^*-/-*^ NGFR Trib2 were treated with DNR and apoptosis and cell cycle profiles were assessed. Our results showed that reintroduction of Trib2 in the *Trib2*^*-/-*^ NH9 cells increased apoptosis in untreated and DNR treated cells to levels comparable with WT NH9 cells (Fig. 7a). NH9-expressing *Trib2*^*-/-*^ NGFR Trib2 cells showed a higher proportion of cells in G0 and less cells in G1 compared to *Trib2*^*-/-*^ NGFR (Fig. 7b). This was supported by the significant decrease in the percentage of p-HH3+ cells in NH9-expressing *Trib2*^*-/-*^ NGFR Trib2 treated cells compared to NH9-expressing *Trib2*^*-/-*^ NGFR treated cells (Fig. 7c). Together, these results demonstrate that Trib2 expression is required for the appropriate cell cycle stress response and the effective killing of myeloid leukaemia cells.

### Pharmacological activation of p38 reverts the drug resistant phenotype of Trib2-deficient myeloid leukaemia cells

**Fig. 8:**
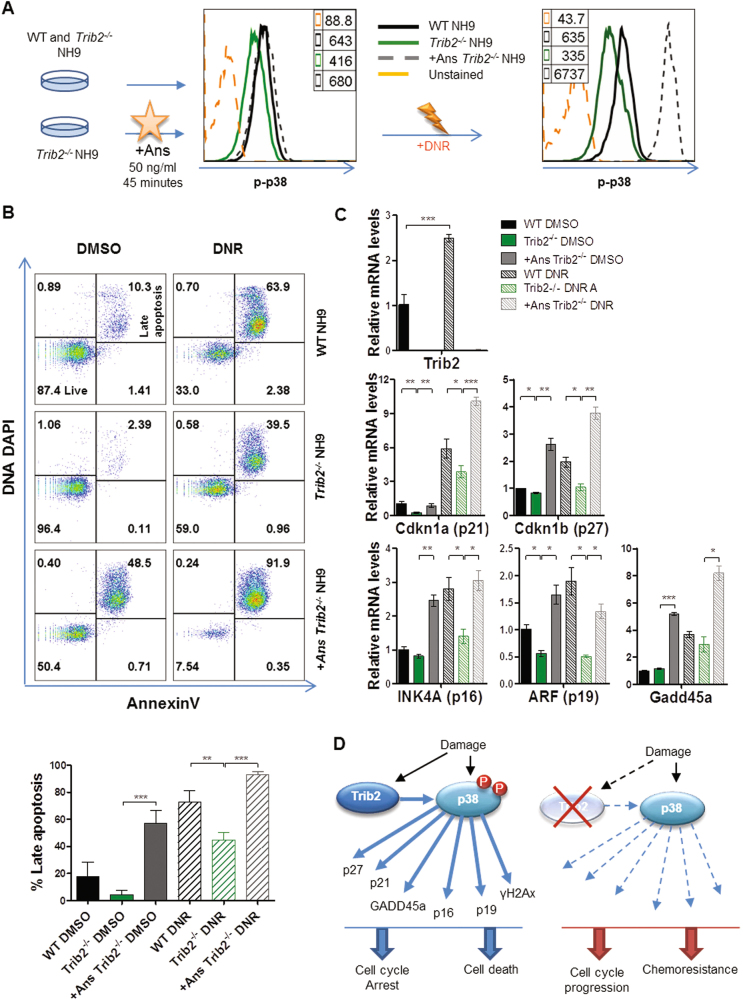
Pharmacological activation of p38 restores stress response pathways in drug treated *Trib2*^*-/-*^ NH9 cells. **a** Ans/DMSO pre-treatment strategy in WT and *Trib2*^*-/-*^ NH9 cells, followed by DNR treatment. Representative histogram of p-p38 expression following Ans pre-treatment and after DNR treatment are shown and MFIs are indicated. **b** Flow cytometric analysis of apoptotic levels by means of AnnexinV/DNA DAPI after 24 h DNR treatment (top panel) and graph percentages of Late Apoptotic (AnnexinV+/DNA DAPI+) cells (bottom panel). Data are representative of 3 independent experiments, graphs show mean ± SD. **P < 0.005, ***P < 0.001, using unpaired *t*-test. **c** *Trib2*, *Cdkn1a* (p21), *Cdkn1*b (p27), *INK4A* (p16), *ARF* (p19), and *Gadd45a* relative mRNA levels in WT, *Trib2*^*-/-*^ NH9 cells and +Ans *Trib2*^*-/-*^ NH9 cells after 16 h DNR treatment. Data are representative of 2 independent experiments with similar trend, graphs show mean of technical replicates ± SD. *P < 0.05, **P < 0.005, ***P < 0.001 using unpaired *t*-test. **d** Schematic representation of the Trib2-p38 regulatory axis. Trib2 promotes damage response pathways via p38 activation (left), and its absence results in impaired signalling response, uncontrolled cell cycle progression and chemoresistance (right)

Next we tested whether pharmacological activation of p38 by Ans could revert the DNR resistant phenotype of the *Trib2*^*-/-*^ NH9 cells similar to Trib2 re-expression (Fig. 8a). Ans pre-treatment resulted in increased basal and DNR-induced apoptosis in the *Trib2*^*-/-*^ NH9 cells (+Ans *Trib2*^*-/-*^ NH9) (Fig. 8b top and bottom panels). In addition, Ans pre-treatment restored drug-induced expression of p38 target genes in the *Trib2*^*-/-*^ NH9 cells to levels comparable (*INK4A*, *ARF*) or higher (*Cdkn1a*, *Cdkn1b*, *Gadd45a*) than those of the WT NH9 treated cells (Fig. 8c). Overall, these data suggest that TRIB2 controls stress signalling responses via p38 activation. We next tested the direct binding of Trib2 and p38 as a mechanism for Trib2-mediated p38 regulation. Using a co-immunoprecipitation (co-IP) assay in Hek293T cells overexpressing myc-tagged Trib2 (PHMA Trib2) or transfected with the empty control plasmid (PHMA) (S6), we did not detect any binding between p38 and Trib2. This result does not exclude the possibility that p38 MAPK and its upstream MAPKK activators could interact with Trib2 in myeloid leukaemic cells transiently under stress conditions.

## Discussion

In this study, we investigated the impact of Trib2 deficiency in myeloid leukaemia. We demonstrate that Trib2 is not required for the initiation of myeloid leukaemia, but is required for p38 and stress signalling, induction of cell cycle checkpoint response and apoptosis. This tumour suppressive role of the TRIB2-p38 axis is also supported by the evidence that Trib2 deficiency accelerates the onset of acute lymphoid leukaemia^12^. We show here the defective stress response manifests a chemotherapy resistant phenotype and a growth advantage to myeloid leukaemia revealing, for the first time, TRIB2 as a tumour suppressor in myeloid leukaemia.

p38 is a regulator of key cell cycle regulators (p21, p16, p19, GADD45), signalling molecules (Chk1, γH2Ax), TFs (Egr1, Fos and Jun) which are inappropriately activated in Trib2 deficient leukaemia cells. These, and others, are involved in proliferation, survival and stress response pathways^38–41^, including the phosphatase CDC25(Ref^7^), which is known to be regulated by Trib2. Therefore, impaired p38 activation due to the absence of Trib2 most likely accounts for the defective signalling response and chemoresistant phenotype of myeloid leukaemia cell (Fig. 8d). Indeed, using IL3 stimulation and the p38 pharmacological activator Ans, p38 phosphorylation/activation was still lower in Trib2 deficient cells compared to treated WT NH9 cells, demonstrating that Trib2 functions in the activation of p38. The re-expression of Trib2 or the activation of p38 in DNR treated Trib2 deficient cells further confirms the Trib2-p38 axis as a regulator of cell cycle progression and damage signalling pathways. As it is a pseudokinase, Trib2 would not lead to phosphorylation of p38 upon binding (whether indirect or direct), but act as a kinase inhibitor/competitive inhibitor/allosteric regulator. It would be interesting to obtain structural data on Trib2 necessary to support for this. To date, no structural data for Trib2 is available.

Our data is consistent with the finding that Trib2 deletion does not impede MLL/AF9-driven myeloid leukaemia^17^, as we did not observe any defect in the ability of NH9 to transform Trib2 deficient cells. Hoxa9-dependent transactivation of the *Trib2* promoter was shown to be efficient only in the presence of the cofactors Meis1 and Pbx3^23,24^. Overexpression of NH9, enhancing self-renewal potential of normal cells, did not induce *Trib2* gene upregulation in WT HSPCs. Moreover, no differences were observed in *Hox* genes expression in presence or absence of Trib2, suggesting that Trib2 cooperativity in myeloid leukaemia^18^ is independent on the HOX transcriptional program. This is supported by the observation that cooperating lesions in myeloid leukaemia usually affect non-redundant pathways, e.g., proliferation/survival and self-renewal/differentiation^42^. Indeed, we have demonstrated that Trib2 modulates the expression or activation of several signalling molecules and cell cycle inhibitors, whereas NH9 confers aberrant self-renewal potential to the cells.

We demonstrated that Trib2 is required in response to stress to efficiently inhibit cell cycle progression and mitosis, in order to prevent the propagation of damaged cells. These data suggest that Trib2 may be an important regulator in myeloid leukaemia drug resistance and relapse. We showed that *Trib2* is transcriptionally upregulated in the WT myeloid leukaemia cells after either starvation or genotoxic challenge. Our results are in line with previous studies, reporting *TRIB2* upregulation in response to survival factor withdrawal in primary T-cells and erythroleukaemia cells^43^, miR155 induction in acute myeloid leukaemia cells^44^ and cisplatin treatment in ovarian cancer cells^6^, which all associated with activation of the apoptotic pathway. Conversely, ectopic or intrinsic high *TRIB2* expression confers chemoresistance in solid cancers and leukaemia, by modulating the AKT/FOXO/p53 signalling pathway^45^ and upregulation of BCL2 expression^46^. Therefore, similar to other renowned cancer related proteins (e.g. E2Fs, p21, GADD45A, MYC and RUNX1), Trib2 obeys the Goldilocks principle in cancer biology; too little or too much are both pro-tumorigenic. Data from us and others would conclude that Trib2 exerts a dual role in myeloid leukaemia and other cancers (e.g., T cell leukaemia, liver), in a stage- and context-specific manner.

Summarising, this work identified a novel Trib2-p38 regulatory pathway important for cell cycle checkpoint and stress responses, with implications on drug resistance and relapse in myeloid leukaemia (Fig. 8d).

## Materials and methods

### Mice

*Trib2* knock out mice (B6; 129S5-Trib2^tm1Lex^, referred to as *Trib2*^*-/-*^), backcrossed onto C57B6 as previously described^12^, were housed in the university of Glasgow. All animals were handled in accordance with good animal practice as defined by the Animals (Scientific Procedures) Act 1986. Mice used in the study were from age matched adult (7-14 week old) WT and *Trib2*^*-/-*^ mice of either sex.

### Primary cell culture, transduction and generation of WT and *Trib2*^*-/-*^ NH9 cell lines

Retroviral supernatants were generated by transient transfection of Hek293T cells with the retroviral constructs (together with pCMV-Gag-Pol packaging vector and pHIT123 envelope vector) with the calcium-phosphate method and titered with NIH-3T3 cell as previously described^15^. For transduction experiments, total bone marrow cells were isolated and were enriched for c-Kit expression, using anti-CD117 (c-Kit) microbeads and MS MACS columns (Miltenyi Biotec) according to manufacturer’s instructions. c-Kit+ cells are an enriched HSPC population. They were cultured o/n in prestimulation medium (DMEM, 15% FBS, 2 mM l-glutamine, 100 U/mL Penicillin/Streptomycin (Pen/Strep), 10 ng/ml IL3, 10 ng/ml IL6, 100 ng/ml SCF). WT and *Trib2*^*-/-*^ HSPCs were transduced with MigR1 or MigR1 NH9 GFP tagged retroviral vectors^31^. WT and *Trib2*^*-/-*^ NH9 immortalized lines were generated by expansion in liquid culture (LC) in prestimulation medium for 9 weeks. Afterwards, the culture was continued in NH9 medium (DMEM, 10% FBS, 10% WEHI-3B conditioned medium, 2 mM l-glutamine, 100 U/mL Pen/Strep). NH9 samples outgrew MigR1 controls and 100% GFP + NH9 cells were immortalised. The growth of the NH9 cell lines was monitored by trypan blue counts. The cells were routinely tested for mycoplasma contamination before and after freezing and thawing. For growth factor deprivation (GFD) experiments, NH9 cell lines were seeded at 0.1 × 10^6^ cells/ml 24 h before starvation. The cells were incubated in starvation medium (DMEM, 2 mM l-glutamine, 100 U/ml Pen/Strep), for the indicated time points. For daunorubicin (DNR) and anisomycin (Ans) treatment experiments, NH9 cell lines were seeded at 0.2 × 10^6^ cells/ml 24 h before treatment with either 50 nM DNR or 25 and 50 ng/ml Ans or DMSO as vehicle control. The DNR inhibitory concentration 50 (IC50) was determined in WT NH9 overexpressing cells (46.07 nM) (S3A). Cells transductions were performed centrifuging 2–5 × 10^6^ cells/ml cells with the required vectors for 90 min at 1290x*g* in either prestimulation medium (HSPCs) or NH9 medium (NH9 cell lines), supplemented with 4 µg/ml of polybrene.

### Colony forming cell (CFC) assay

For CFC assays of freshly transduced HSPCs (isolated by c-Kit enrichment), equal numbers of sorted GFP-expressing cells (0.5 or 10 × 10^3^) were seeded in MethoCult™ GF M3434 (Stem Cell Technologies) (MC) medium, containing 15% FBS, 1% BSA, insulin (10 μg/ml), transferrin (200 μg/ml), IL3 (10 ng/ml), IL6 (10 ng/ml), SCF (50 ng/ml) and Erythropoietin (3 U/mll), and supplemented with Pen/Strep (100 U/ml). For DNR wash out CFC assays, equal numbers of WT and *Trib2*^*-/-*^ NH9 cells (0.1 or 30 × 10^3^) were treated with DNR for 24 h and remaining cells plated in MC medium (M3231, Stem Cell Technologies), containing 30% FBS and 1% BSA, supplemented with IL3 (10 ng/ml) and Pen/Strep 100 U/ml. Colonies were scored after 1 to 2 weeks.

### Fluorescent activated cell sorting (FACS) analyses

Flow cytometry experiments were performed using BD FACSCanto™ II and BD FACSAria™ (BD biosciences, UK). Cell trace™ violet (CTV, Invitrogen) staining was performed following manufacturer instructions and the fluorescent signal assessed in the proliferating samples after 4 days of culture. Cells treated for 24 h with 100 ng/ml demecolcine solution (Sigma) were used as undivided control (colcemid control) and used to calculate the undivided generation peak (division 0). Apoptosis was measured by AnnexinV (eBioscience) / 4′,6-diamidino-2-phenylindole (DAPI, Sigma) staining, performed in HBSS solution (Gibco). For staining of the intracellular antigens, the cells of interest were fixed and permeabilised using the BD Cytofix/Cytoperm™ Fixation/Permeabilization Kit (BD biosciences), subsequently incubated with anti-Ki67 (eBioscience), anti-phospho(p)-p38 (eBioscience), anti-phospho(p)-Chk1 (New England Biolab) or anti-γH2Ax (BD Biosciences) antibodies (Table S1). For mitotic index measurement, the cells were fixed using the BD Fixation/Permeabilization solution (BD biosciences) and permeabilised with 90% methanol, followed by incubation with anti-phospho-Histone H3 (p-HH3) antibody (Cell Signaling Technology, Table S1). DNA staining was performed on fresh or fixed samples using PI/RNase Staining Solution (BD biosciences), or DAPI, for cell cycle or viability analyses. For surface antigens detection, fresh samples were incubated with anti-CD3, anti-CD4, anti-CD8, anti-CD19, anti-B220, anti-Ter119, anti-Gr1, anti-CD127 (IL7R), anti-CD34, anti-CD16/32, anti-CD11b, anti-CD117(c-Kit) and anti-Sca1 antibodies (eBioscience), and DAPI+ dead cells exclusion was performed. In all flow cytometry analyses, the desired populations were gated through FSC-Area/SSC-Area and doublets were excluded based on FSC-Height/FSC-Area.

### Real time PCR (RT-PCR)

Up to 1 µg of total RNA was reverse transcribed with the high capacity cDNA reverse transcription kit (Applied Biosystems), following manufacturers’ instruction. In case of low cell number samples, Specific Target Amplification (STA) reaction was performed with the Qiagen® Multiplex PCR Kit (Qiagen), following manufacturers’ instructions. RT-PCR was performed using the Fast SYBR™ Green 2× Master Mix (Applied Biosystems), according to manufacturers’ instruction. All primers, listed in Table S2, were designed to target murine sequences. Failed reactions/outliers were excluded from the analyses.

### Co-immunoprecipitation (Co-IP)

Hek293T cells were transiently transfected with the myc-tagged PHMA Trib2 or PHMA control vectors as previously described^15^. The transfected cells were harvested at 24 h and whole cell lysates were prepared using ice-cold Hepes buffer (50 mM Hepes pH 7.4, 150 mM NaCl, 1 mM EDTA, 0.5% NP-40, 5% glycerol, with protease and phosphatase inhibitors). Crosslinking was performed using dithiobis-succinimidylpropionate (DSP) (Thermo Fisher Scientific) at a concentration of 1.5 mM, and the reaction quenched using Tris (pH 7.4) (50 mM). One milligram of precleared lysates were incubated with Myc9E10 antibody (Santa Cruz Biotechnology) or normal mouse IgG overnight at 4 °C, followed by incubation with Protein A/G UltraLink™ Resin (Thermo Fisher Scientific) for 1.5 h at 4 °C. The samples were washed in Tris buffer (50 mM Tris pH 7.4, 150 mM NaCl, 1 mM EDTA, 0.5% NP-40, 5% glycerol, with protease and phosphatase inhibitors), eluted in Laemmli buffer and analysed by western blotting.

### Western Blotting

Whole cell lysates were prepared using ice-cold modified RIPA buffer (50 mM Tris, pH 8.0, containing 0.5% NP-40, 0.25% sodium deoxycholate, 150 mM NaCl, 1 mM EDTA, with protease and phosphatase inhibitors). Up to 25 µg of protein samples were resolved on hand-casted SDS-PAGE gels, transferred to 0.45 μm nitrocellulose membrane (Whatman) and analysed by immunoblotting with the antibodies listed in Table S3. SuperSignal™ West Pico or Femto Chemoluminescent Substrates (Thermo Fisher Scientific) were used for signal detection on CL-XPosure™ radiography films. The films were developed using the Medical film processor SRX-101A (Konika Minolta, Tokyo, Japan).

### Metaphase spreads

WT and *Trib2*^*-/-*^ NH9 cells were seeded at 0.2 × 10^6^ cells/ml and treated with DNR/ DMSO from WT and *Trib2*^*-/-*^ NH9 cells after 24h DNR treatment from WT and *Trib2*^*-/-*^ NH9 cells after 24h DNR treatment for 24h. Demecolcine solution (Sigma) was added to the cell suspension (100 ng/ml) 45 min prior the end of the treatment. The cells were incubated at 37 °C for 15 min in 75 mM KCl (swelling). The cell were fixed in 3:1 methanol:acetic acid. Fixed cells were dropped on microscope slides and let dry at RT. Mounting medium with DAPI (Vector shield) was applied to each sample. The slides were examined at an Axio Imager M1 Epifluorescence and Brightfield Microscope (Zeiss) and pictures captured to show representative mitotic cells (Axio Vision software).

### Statistics

Graphs and statistical analyses were performed using GraphPad Prism 5 (GraphPad Software, La Jolla California USA). Flow cytometry data were analysed using FlowJo (Tree Star, Ashland OR USA). Unpaired, two-tailed Student’s *t*-test was used to test statistical significance whenever comparing two experimental groups with similar variance. Two-way ANOVA was used to evaluate the effect of the genotype (WT and *Trib2*^*-/-*^) on the fold change p-p38 expression over two doses of Ans stimulation. The statistical test used, the *P* value (*P < 0.05, **P < 0.005, ***P < 0.001) and the number of times each experiment was biologically and technically replicated was indicated in the related figure legends and graphs.

## Electronic supplementary material

Supplementary(DOCX 34 kb)

Supplementary
