# Supplementary material for: A Trib2-p38 axis controls myeloid leukaemia cell cycle and stress response signalling
Source: Cell Death Dis. 2018 Apr 18;9(5):443. doi: 10.1038/s41419-018-0467-3 (PMC5906628; doi:10.1038/s41419-018-0467-3)
Supplement: Supplementary file 1 — Supplementary(DOCX 34 kb) [file 41419_2018_467_MOESM1_ESM.docx]

## Supplementary Figures & Legends

**A Trib2-p38 axis controls myeloid leukaemia cell cycle and stress response signalling**

Trib2 in leukaemia cell cycle and stress response

Mara Salomè^1^, Aoife Magee^1^, Krisha Yalla^1^, Shahzya Chaudhury^1^, Evgenia Sarrou^1^, Ruaidhrí J Carmody^2^ and Karen Keeshan^1^

^1^Paul O’Gorman Leukaemia Research Centre, Institute of Cancer Sciences,

University of Glasgow, Scotland, UK

^2^Centre for Immunobiology, Institute of Infection, Immunity and Inflammation, University of Glasgow, Scotland, UK

Corresponding author:

Dr Karen Keeshan

Paul O’Gorman Leukaemia Research Centre, Institute of Cancer

Sciences, University of Glasgow, Scotland

Tel: 0044 141 301 7895

Email: Karen.keeshan@glasgow.ac.uk

**Figure S1 NH9 can transform WT and Trib2 deficient HSPCs in both CFC and liquid culture conditions:**

A) Schematic CFC assay experimental strategy. WT and *Trib2^-/-^* HSPCs transduced with MigR1 or NH9 vectors were sorted for GFP expression at 48 hours post transduction and serial replating ability assessed through CFC assay. NH9 samples formed colonies up to 4 rounds of CFC and NH9-transformed cells from CFC3 were able to grow in LC conditions. B) Representative histograms showing GFP expression levels at 48 hours post transduction with MigR1 or NH9 vectors in WT and *Trib2^-/-^* HSPCs. C) Representative colony pictures (10X) at CFC3 of WT and *Trib2^-/-^* MigR1 and NH9 samples. NH9 samples formed several big and dense colonies, whereas MigR1 control samples showed few scattered cells. D) Schematic experimental strategy for transformation of WT and *Trib2^-/-^* HSPCs transduced with NH9 vectors in LC conditions and generation of WT and *Trib2^-/-^* NH9 immortalised cell lines. E) Graph shows GFP expression profile of unsorted WT and *Trib2^-/-^* MigR1 or NH9 samples over time.

**Figure S2 Leukaemic Stem Cell stainings**

A) Full gating strategy for staining shown in Figure 1C and D. B) Full gating strategy for staining shown in Figure 1F.

**Figure S3 In the absence of Trib2, AML cells remain in M phase in response to GFD.**

A) Graph shows cell viability of WT NH9 expressing cells in response to increasing concentrations of DNR, as measured by Trypan blue cell counts. In black is projected the non-linear regression curve that best fit the experimental data and the IC50 is indicated. B) Flow cytometric analyses of the mitotic index in WT and *Trib2^-/-^* NH9 cells after 24h GFD, as measured by p-HH3/PI DNA levels. C) Graphed percentages, at the indicated time points, as measured in (B). Data are representative of 3 independent experiments, graphs show mean ±SD. *P<0.05, using unpaired t test.

**Figure S4 Trib2 is required for transcriptional activation of stress signaling mediators and TFs in response to stress conditions.**

A) *Trib2,* *Cdkn1a*(p21), *Cdk4*, *INK4A* (p16) and *ARF* (p19) relative mRNA levels in WT and *Trib2^-/-^* NH9 cells after 0 (basal) and 8 hours of GFD. Data are representative of 2 (p16 and p19) or 3 (Trib2, p21 and Cdk4) independent experiments with similar trend, graphs show mean of technical replicates ±SD. *P<0.05, **P<0.005, ***P<0.001 using unpaired t test. B) Proapoptotic TFs *ATF2,* *Max*, *Egr1*, *Nfatc4*, *Jun* and *Fos* gene expression levels in WT and *Trib2^-/-^* NH9 cells after 16 hours DNR treatment. Graphs show mean of 2 (*Nfatc4* and *Jun*) or 3 (*ATF4*, *Max*, *Egr1*, *Fos*) biological replicates ±SD, generated from 3 independent experiments. *P<0.05, **P<0.005 using unpaired t test. C) MAPK signaling genes *MAPK3* (ERK1), *MAPK9* (JNK2), *MAPK11* (p38β), *MAPK12* (p38γ), *MAPK13* (p38δ), *MAPK14* (p38α) and *MKNK1* (MNK1) relative mRNA levels in WT and *Trib2^-/-^* NH9 cells after 16 hours DNR treatment. Graphs show mean of 2 (*MAPK11*, *MAPK12* and *MAPK13*) or 3 (*MAPK3*, *MAPK9*, *MAPK14*, *MKNK1*) biological replicates ±SD, generated from 3 independent experiments. *P<0.05, **P<0.005, ***P<0.001 using unpaired t test.

**Figure S5 Retroviral expression of *Trib2* rescues the apoptotic phenotype in *Trib2^-/-^* NH9 cells.**

A) Schematic experimental strategy: WT and *Trib2^-/-^* NH9 cells were transduced with MigR1 NGFR and NGFR Trib2 constructs and after 48 hours GFP+NGFR+ cells were FACS sorted and used for downstream analyses. B) Western blot analysis of Trib2 exogenous expression. C) Relative *Trib2* mRNA expression in MigR1 NGFR and NGFR Trib2 transduced cells. Graph shows mean ±SD of technical replicates and is representative of independent transduction experiments.

**Figure S6 Trib2 PHMA does not interact with p38 in Hek293T cells.**

A) Western blot analysis of total p38 and Myc9E10 in IP Myc9E10 and IgG control samples, from PHMA and PHMA Trib2 overexpressing Hek293T. B) Western blot analysis of total input lysates shows levels of protein expression. Data is representative of 2 independent experiments.

## Supplementary Figures


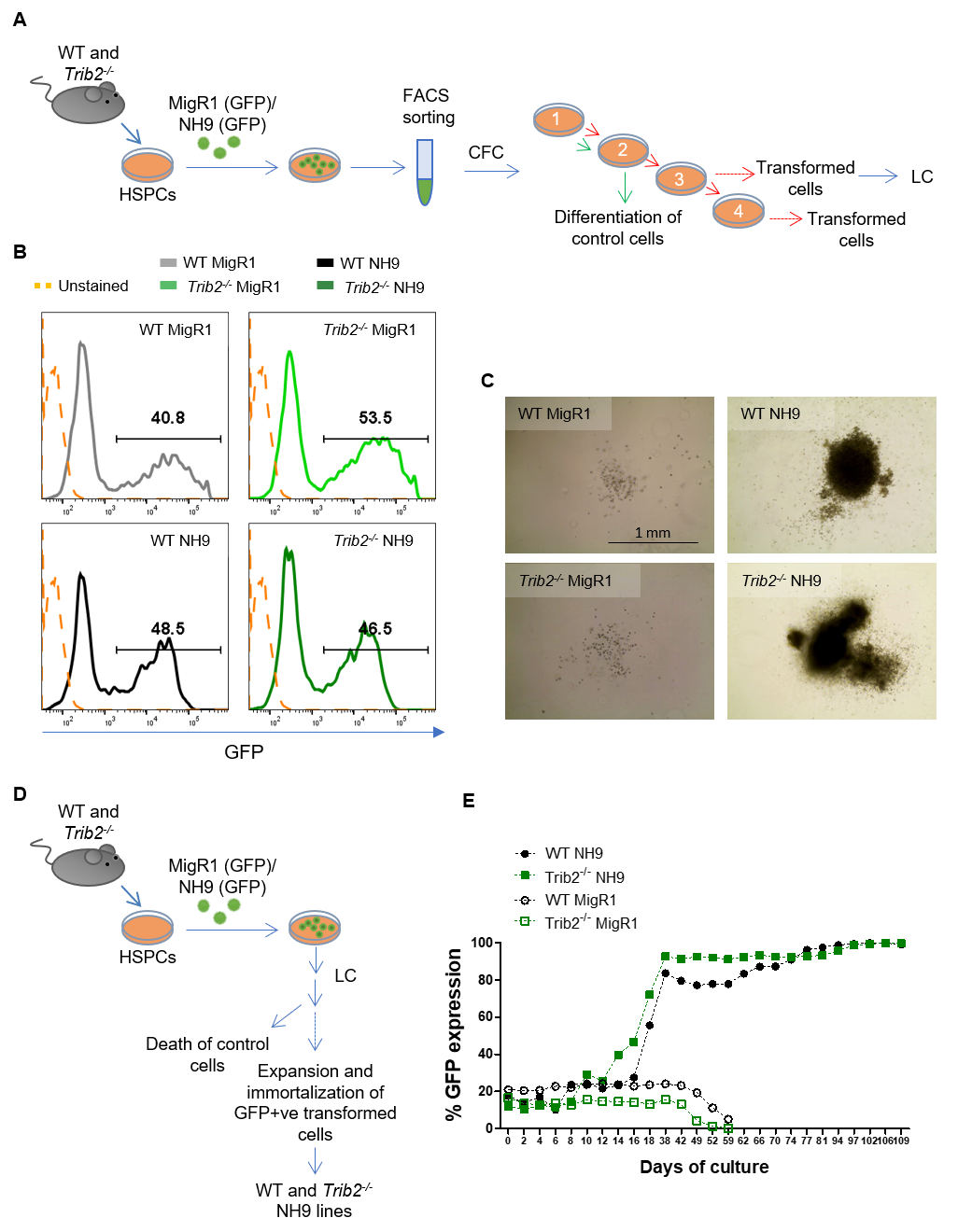


Figure S1


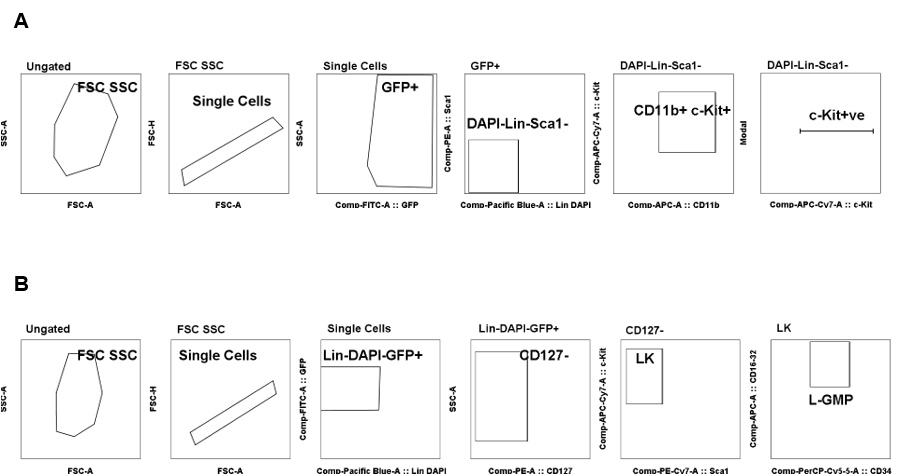


Figure S2


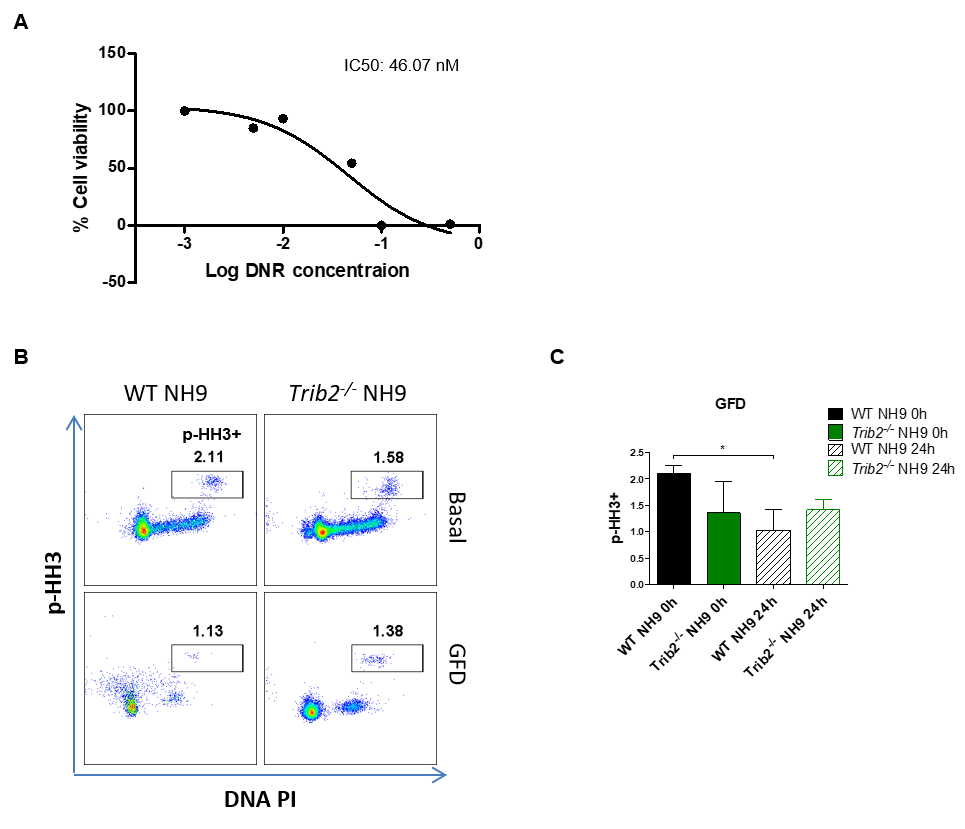


Figure S3


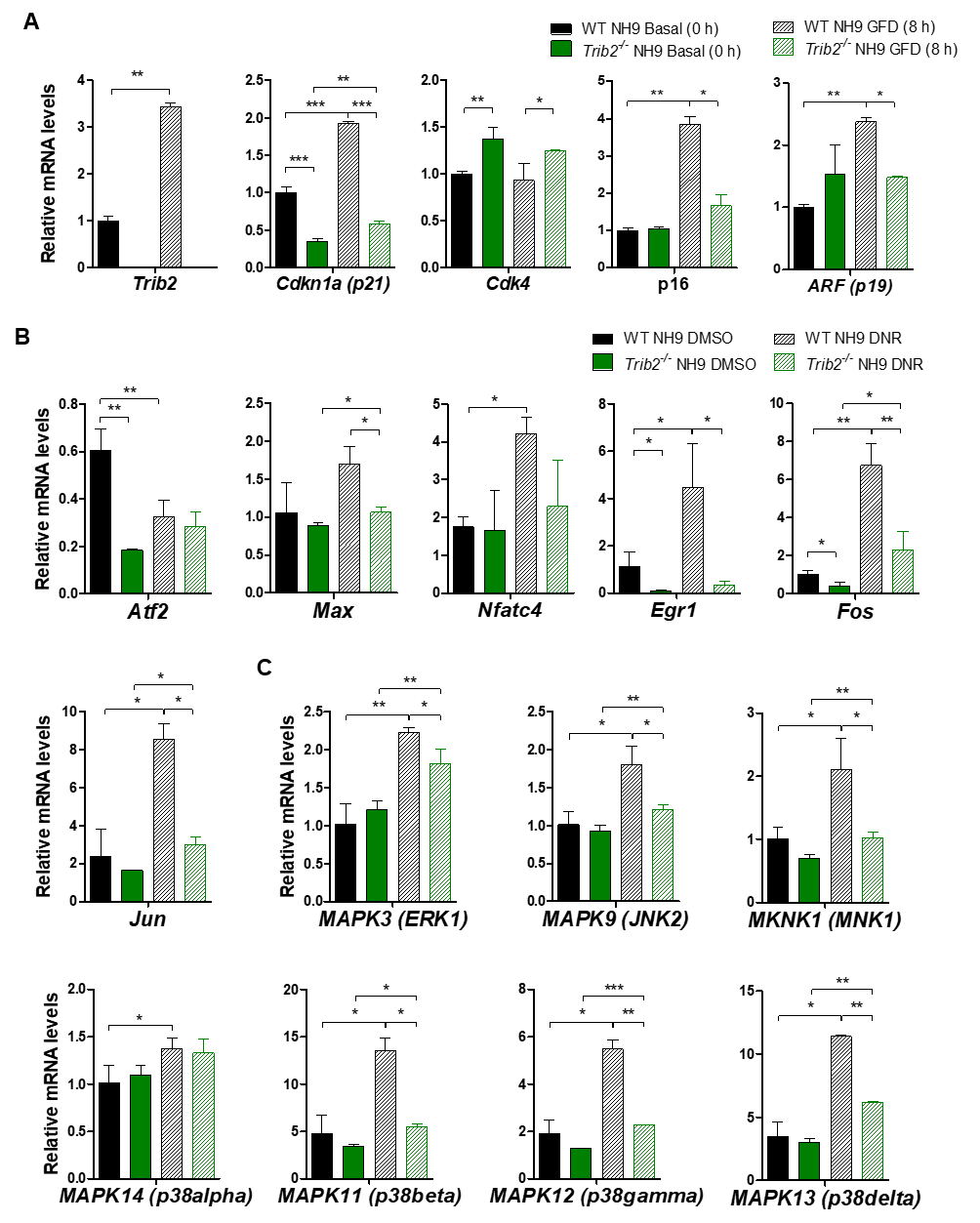


Figure S4


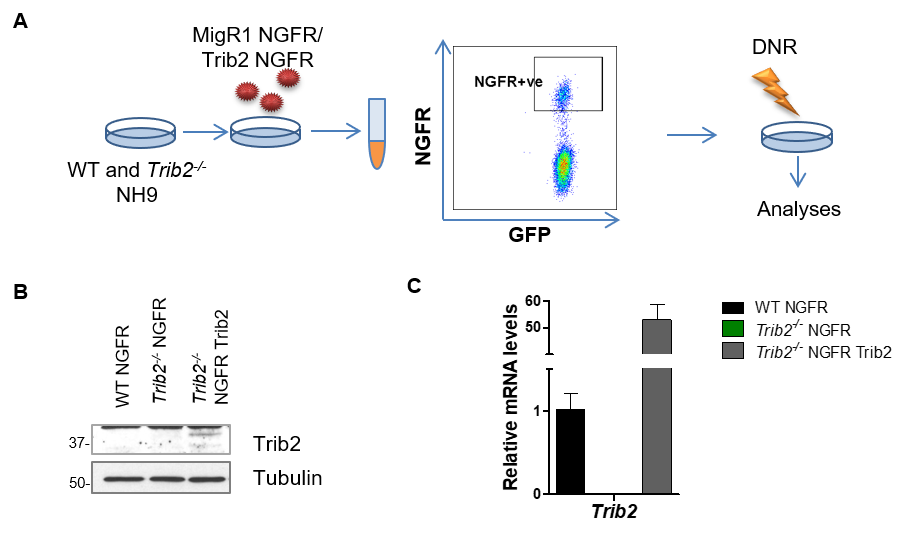


Figure S5


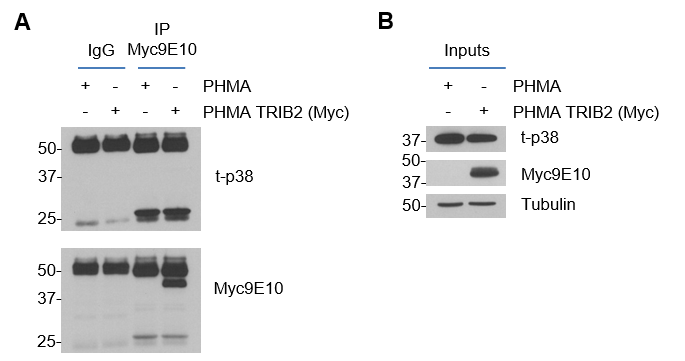


Figure S6
