# Supplementary material for: A Trib2-p38 axis controls myeloid leukaemia cell cycle and stress response signalling
Source: Cell Death Dis. 2018 Apr 18;9(5):443. doi: 10.1038/s41419-018-0467-3 (PMC5906628; doi:10.1038/s41419-018-0467-3)
Supplement: Supplementary file 2 — Supplementary [file 41419_2018_467_MOESM2_ESM.docx]

**Supplementary Tables**

Mara Salomè^1^, Aoife Magee^1^, Krisha Yalla^1^, Shahzya Chaudhury^1^, Evgenia Sarrou^1^, Ruaidhrí J Carmody^2^ and Karen Keeshan^1^

^1^Paul O’Gorman Leukaemia Research Centre, Institute of Cancer Sciences,

University of Glasgow, Scotland, UK

^2^Centre for Immunobiology, Institute of Infection, Immunity and Inflammation, University of Glasgow, Scotland, UK

Corresponding author:

Dr Karen Keeshan

Paul O’Gorman Leukaemia Research Centre, Institute of Cancer

Sciences, University of Glasgow, Scotland

Tel: 0044 141 301 7895

Email: Karen.keeshan@glasgow.ac.uk

| **Antibody/ reagent** | **Clone** | **Format** | **Manufacturer** | **Type of Staining** | **Catalog number** |
| --- | --- | --- | --- | --- | --- |
| **CD3** | 145-2C11 | eFluor450 | eBioscience | Surface | 48-0031-82 |
| **CD4** | RM4-5 | eFluor450 | eBioscience | Surface | 48-0042-82 |
| **CD8** | 53-6.7 | eFluor450 | eBioscience | Surface | 48-0081-82 |
| **CD19** | eBio1D3 | eFluor450 | eBioscience | Surface | 48-0193-82 |
| **B220** | RA3-6B2 | eFluor450 | eBioscience | Surface | 48-0452-82 |
| **Ter119** | Ter119 | eFluor450 | eBioscience | Surface | 48-5921-82 |
| **CD127 (IL7R)** | SB/199 | PE | BD Biosciences | Surface | 552543 |
| **CD34** | RAM34 | Biotin | eBioscience | Surface | 13-0341-82 |
| **CD16/32** | 93 | APC | eBioscience | Surface | 17-0161-81 |
| **Streptavidin** |  | PercPCy5-5 | eBioscience | Surface | 45-4317-82 |
| **CD11b** | M1/70 | APC | eBioscience | Surface | 17-0112-83 |
| **c-Kit (CD117)** | 2B8 | APC-eFluor780 | eBioscience | Surface | 47-1171-82 |
| **Sca1** | D7 | PE-Cy7/PE | eBioscience | Surface | 25-5981-82/ 12-5981-83 |
| **p-HH3 (S10)** | D2C8 | n/a | Cell Signaling Technologies | Intracellular | 9701 |
| **anti-Rabbit IgG** | Polyclonal (A-31556) | AlexaFluor 405 | Thermo Fisher Scientifics | Intracellular (Secondary) | A31556 |
| **Ki-67** | SolA15 | PE-Cy7 | eBioscience | Intracellular | 25-5698-82 |
| **p-p38 (T180/Y182)** | 4NIT4KK | APC | eBioscience | Intracellular | 17-9078-41 |
| **p-Chk1 (S345)** | 133D3 | PE | Cell Signaling Technologies | Intracellular | 12268S |
| **γH2aX (S139)** | N1-431 | PerCP-Cy5.5 | BD Biosciences | Intracellular | 564718 |
| **CTV** |  |  | Invitrogen | Cell Divsion | C34557 |
| **Annexin V** |  | PE | eBioscience | Apoptosis | 17-8007-74 |

Table S1: FACS antibodies and reagents

| **ID** | **Gene name** | **Forward primer** | **Reverse primer** |
| --- | --- | --- | --- |
| **Atf2** | Activating Transcription factor 2 | TGTGGCCAGCGTTTTACCAA | TGTTGGCGTTGGAGTCTGAT |
| **Bmi1** | BMI1 Proto-Oncogene | ATACCTGGAGAAGAAATGGCCC | CAGCTCTCCAGCATTCGTCA |
| **Cdk4** | Cyclin Dependent Kinase 4 | CGGCCTGTGTCTATGGTCTG | CTCGAAGCAGGGGATCTTACG |
| **Cdkn1a (p21)** | Cyclin Dependent Kinase Inhibitor 1A | TCCCACTTTGCCAGCAGAATA | CACGGGACCGAAGAGACAAC |
| **Cdkn1b (p27)** | Cyclin Dependent Kinase Inhibitor 1B | TTCGCAAAACAAAAGGGCCAA | ATTCTTAATTCGGAGCTGTTTACG |
| **Egr1** | Early Growth Response 1 | CCTGACCACAGAGTCCTTTTC | AGCGGCCAGTATAGGTGATG |
| **Fos** | Fos Proto-Oncogene | TCCAAGCGGAGACAGATCAAC | CGGTGGGCTGCCAAAATAAA |
| **Gadd45A** | Growth Arrest And DNA Damage Inducible Alpha | GGTGACGAACCCACATTCAT | ACCCACTGATCCATGTAGCG |
| **Gadd45B** | Growth Arrest And DNA Damage Inducible Beta | CTCCTGGTCACGAACTGTCA | TGGGTCTCAGCGTTCCTCTA |
| **GusB** | Glucuronidase Beta | GGGACAAAAATCACCCTGCG | GCGTTGCTCACAAAGGTCAC |
| **HoxA7** | Homeobox A7 | CGCCTCCTACGACCAAAACA | CTTCCTGTCGGGTCCTGAAC |
| **HoxA9** | Homeobox A9 | CCCTGACTGACTATGCTTGTGGT | TCTCCGCCGCTCTCATTCTC |
| **Jun** | Jun Proto-Oncogene | CAAGATGGACTGGGTTGCGA | CAAAGTCTGCCGGCCAATAG |
| **Mapk3 (ERK1)** | Extracellular signal-regulated kinases 1 | GTACGGCATGGTCAGCTCAG | ATCTGGATCTCCCTCAGCGT |
| **Mapk9 (JNK2)** | C-Jun N-Terminal Kinase 2 | CGATTGAAGAGTGGAAAGAGCTAA | CTGAAGGCTGGTCTTTTACCC |
| **Mapk11 (p38β)** | Mitogen-Activated Protein Kinase p38 Beta | AAGCCAGTGTCCCTAA | CCACAGGCAACCACAAATCT |
| **Mapk12 (p38γ)** | Mitogen-Activated Protein Kinase p38 Gamma | GATGAACCCAAGGCCCAGAA | CGCTTCCATTCCTCAAGGGT |
| **Mapk13 (p38δ)** | Mitogen-Activated Protein Kinase p38 Delta | GCTCACCCCTTCTTTGAACC | TTCGTCCACGCTGAGTTTCT |
| **Mapk14 (p38α)** | Mitogen-Activated Protein Kinase p38 Alpha | AAGACTCGTTGGAACCCCAG | TCCAGTAGGTCGACAGCCAG |
| **Max** | MYC Associated Factor X | GAGGTTTCAATCTGCGGCTG | TGAGTCCCGCAAACTGTGAA |
| **Meis** | Myeloid Ecotropic Viral Integration Site 1 | CAGTCCAACCGAGCAGTCAG | CTGGCATACTTTGCAGCCCT |
| **Mknk1 (Mnk1)** | MAP Kinase Interacting Serine/Threonine Kinase 1 | CCGCGGTGGTCAACTAAGAT | CACAGAGATGGAACACAATGGG |
| **Nfatc** | Nuclear Factor Of Activated T-Cells 4 | CTACAATGAGGTGGGGCCAG | TCTCACTCACTTCCTCCAGGG |
| **p16INK4A** | CDK4 Inhibitor P16-INK4 | ATACCTGGAGAAGAAATGGCCC | CAGCTCTCCAGCATTCGTCA |
| **p19ARF** | p19 Alternate open Reading Frame | GGTGAAGTTCGTGCGATCC | CGTGAACGTTGCCCATCATC |
| **Rnf20** | Ring Finger Protein 20 | ACCATCAATGCCCGGAAGTT | GCAGCGATACTCTGGGGTTT |
| **Runx1 (Aml1)** | Runt Related Transcription Factor 1 | AGGCAGGACGAATCACACTG | CTCGTGCTGGCATCTCTCAT |
| **Smad7** | SMAD Family Member 7 | CCTCGGAAGTCAAGAGGCTG | CAGCCTGCAGTTGGTTTGAG |
| **Sox4** | Sex Determining Region Y-Box 4 | GGTTTCCAGTTCTTGCACGC | TGCAACAGACCGGCATGAAT |
| **Trib2** | Tribbles Homolog 2 | AGCCCGACTGTTCTACCAGA | AGCGTCTTCCAAACTCTCCA |
| **Trib2 (Lexco 1136)** | Tribbles Homolog 2 | CACAATAGCGAGATATGGGAG | GCAATGCGACAAGTTCGGAG |

Table S2: List of primers

| **Antibody** | **Source** | **Clone** | **Manufacturer** | **Catalog number** |
| --- | --- | --- | --- | --- |
| **α-Tubulin** | Mouse | B-5-1-2 | Sigma | T5168 |
| **β-Actin** | Mouse | AC-15 | Sigma | A5441 |
| **p-ERK1-2 (T202/Y204)** | Rabbit | D13.14.4E | Cell Signaling Technologies | 4370 |
| **t-ERK1-2** | Rabbit | 137F5 | Cell Signaling Technologies | 4695 |
| **p-p38 (T180/Y182)** | Rabbit | D3F9 | Cell Signaling Technologies | 4511 |
| **t-p38** | Rabbit | D13E1 | Cell Signaling Technologies | 8690 |
| **p-JNK (T183/Y185)** | Rabbit | Polyclonal (9251) | Cell Signaling Technologies | 9251 |
| **JNK1** | Mouse | 2C6 | Cell Signaling Technologies | 3708 |
| **p-Akt (S473)** | Rabbit |  | Cell Signaling Technologies | 9271 |
| **Pan-Akt** | Mouse | 40D4 | Cell Signaling Technologies | 3653 |
| **Trib2** | Mouse | B-06 | Santa Cruz | SC-100878 |
| **p21** | Rabbit | C-19 | Santa Cruz | SC-397 |
| **Myc9E10** | Mouse | 9E10 | Santa Cruz | SC-40 |
| **Normal Mouse IgG** | Mouse |  | Santa Cruz | SC-2025 |
| **Anti-mouse IgG HRP-linked** | Sheep | Polyclonal (NXA931) | GE-Healthcare | NXA931 |
| **Anti-rabbit IgG HRP-linked** | Donkey | Polyclonal (NA934) | GE-Healthcare | NA934 |

Table S3: List of WB antibodies
